# Sowing Methods and Strigolactones Alleviate Damage to the Photosynthetic System of Rice Seedlings Under Salt Stress by Enhancing Antioxidant Capacity

**DOI:** 10.3390/antiox14081020

**Published:** 2025-08-20

**Authors:** Shaobiao Duan, Liming Zhao, Weinan Chen, Qicheng Zhang, Jiangyuan Ya, Wenji Zhong, Qianqian Shang, Jinji Tu, Hongtao Xiang, Jianqin Zhang, Junhua Zhang

**Affiliations:** 1 College of Coastal Agriculture, Guangdong Ocean University, Zhanjiang 524088, China; 16673903587@stu.gdou.edu.cn (S.D.); shaoguoyang@stu.gdou.edu.cn (W.C.); 13969389281@stu.gdou.edu.cn (Q.Z.); 17878818934@stu.gdou.edu.cn (J.Y.); 18288676490@stu.gdou.edu.cn (W.Z.); 13201398521@stu.gdou.edu.cn (Q.S.); 15798041518@stu.gdou.edu.cn (J.T.); 21122040151@stu.gdou.edu.cn (J.Z.); 18378530354@stu.gdou.edu.cn (J.Z.); 2 Suihua Branch of Heilongjiang Academy of Agricultural Machinery Engineering Sciences, Suihua 152000, China; xianght@163.com

**Keywords:** rice, seeding methods, GR24, salt stress, antioxidant capacity, ionic homeostasis, endogenous hormones, ultrastructure of chloroplasts

## Abstract

Seedling cultivation of rice (*Oryza sativa* L.) is a critical initial step in rice production. This study investigated the effects of sowing methods and strigolactone (GR24) on rice seedlings under salt stress. Results showed that drill-sown seedlings exhibited superior quality under normal conditions compared to broadcast-sown seedlings. Salt stress significantly increased the contents of Cl^−^, Na^+^, reactive oxygen species (ROS), and malondialdehyde (MDA), disrupted chloroplast structure and hormonal balance, and reduced gas exchange parameters and chlorophyll fluorescence parameters. Notably, drill-sowing conferred stronger salt tolerance than broadcast-sowing. Exogenous application of GR24 enhanced activities of antioxidant enzymes—including superoxide dismutase (SOD), ascorbate peroxidase (APX), peroxidase (POD), and catalase (CAT)—and elevated non-enzymatic antioxidant contents such as ascorbic acid (ASA), glutathione (GSH), total phenolics, and flavonoids, alongside related enzyme activities. Concurrently, GR24 reduced Na^+^ and Cl^−^ accumulation, lowered the Na^+^/K^+^ ratio, and increased the contents of K^+^, Ca^2+^, Mg^2+^, and hormones. Consequently, GR24 decreased MDA and ROS levels, protected membrane integrity, reduced electrolyte leakage, repaired chloroplast structure, and improved gas exchange and chlorophyll fluorescence parameters. Due to their superior spatial distribution and photosynthetic efficiency, drill-sown seedlings synergized with GR24 to enhance antioxidant capacity under salt stress, enabling more effective scavenging of peroxidative radicals, stabilization of the photosynthetic system, and mitigation of salt-induced growth inhibition. Ultimately, this combination demonstrated greater stress alleviation than broadcast-sown seedlings.

## 1. Introduction

Rice (*Oryza sativa* L.) is the world’s most important food crop and is highly sensitive to salinity [1]. Currently, rice seedling cultivation primarily employs drill seeding and broadcast seeding. Broadcast seeding results in uneven seed distribution, which hinders the establishment of high-yielding plant populations [2]. In contrast, drill seeding achieves uniform seed distribution in seedling trays, enhancing the uniformity of seedling arrangement and overall seedling quality [3]. The saline–alkali soil in China is constantly expanding, and its total area currently ranks third in the world [4]. Guangdong has extensive areas of saline soil—coastal solonchak. The coastal solonchak area in Guangdong Province covers 10.71 × 10^4^ hm^2^, accounting for 0.73% of the province’s total soil area [5]. Coastal solonchak is formed through the flocculation and deposition of sediments carried by surface runoff into the sea or shallow-sea deposits stirred by waves, which accumulate in the intertidal zone under the action of tides and currents, gradually elevating the tidal flat until it emerges above sea level [6]. Coastal solonchak represents one of the primary types of low-to-medium-yield farmland, characterized by poor soil physicochemical properties, severe surface salt accumulation, and high water evaporation. Currently, the mutual encroachment between construction land and agricultural land, driven by economic development, is increasingly severe. Reclaiming coastal solonchak to create new “granaries” by claiming land from the sea is a crucial approach to alleviating agricultural land conflicts, ensuring food security, and transforming agricultural development models [7]. Salt stress impacts plants through ion toxicity and osmotic stress [1]. Excessive accumulation of Na^+^ and Cl^−^ ions in cells reduces cellular osmotic potential, causing plant dehydration [8]. The excessive buildup of reactive oxygen species (ROS) induces lipid peroxidation, damaging membrane structure and permeability [9]. Furthermore, under saline conditions, stomatal closure in plants restricts CO_2_ supply, impairing photosynthetic capacity and ultimately reducing plant biomass [10].

Strigolactones (SLs) are plant hormones intensively studied in recent years. Current research on the physiological effects of SLs in plants typically employs the synthetic compound GR24 for exogenous application. GR24 has been validated as an effective SL analogue [11] and is widely investigated in the context of abiotic stress responses. Under salt stress, SL application enhances the ROS scavenging capacity, thereby mitigating cellular damage. In salt-stressed rapeseed (*Brassica napus*), GR24 treatment alleviated the decline in photosynthetic efficiency and upregulated the activities of peroxidase (POD) and superoxide dismutase (SOD), while reducing lipid peroxidation [12]. In grapevine (*Vitis vinifera*) seedlings, GR24 effectively induced stomatal closure, modulated chlorophyll composition, counteracted drought-induced photosynthetic inhibition, and activated the ROS scavenging system. Additionally, it elevated ABA levels [13]. Exogenous GR24 mitigated the impact of low-temperature stress on rapeseed seedlings by upregulating antioxidant enzyme-encoding genes, enhancing antioxidant activity, and optimizing photosynthetic pathways [14]. In barley (*Hordeum vulgare*), GR24 increased the levels of reduced glutathione (GSH), ascorbic acid (AsA), and the activity of reductases in the AsA-GSH cycle, while decreasing cadmium (Cd) accumulation [15].

Currently, research on the application of GR24 in rice seedling cultivation under saline–alkali conditions remains limited. We hypothesize that the uptake of GR24 by row-sown seedlings may be enhanced, thereby improving antioxidant capacity and scavenging reactive oxygen species to protect the photosynthetic system under salt stress. This study adopted two sowing methods: drill sowing and broadcast sowing. Rice seedlings were subjected to salt stress and foliar spraying with GR24 at the three-leaf stage. The effects on seedling morphology, photosynthetic apparatus, antioxidant capacity, and hormone levels were analyzed. This research aims to deepen the understanding of the physiological mechanisms underlying GR24-induced salt tolerance, providing a theoretical basis for improving salt tolerance in rice seedlings.

## 2. Materials and Methods

### 2.1. Experimental Materials

The tested rice variety was HuaHang 51, bred by the National Engineering Research Center of Plant Space Breeding and provided by Guangdong Huanong Seed Industry Co., Ltd. (Guangzhou, China). The synthetic strigolactone analogue GR24 was the regulator supplied by Shanghai Macklin Biochemical Technology Co., Ltd. (Shanghai, China). The experimental soil was red soil, with the following physicochemical properties: pH = 7.02, total nitrogen (N) 486 mg·kg^−1^, total phosphorus (P) 372 mg·kg^−1^, and total potassium (K) 227 mg·kg^−1^.

### 2.2. Experimental Methods

This experiment was conducted in a sunlit multi-span greenhouse at the Institute of Agricultural Biotechnology, Guangdong Ocean University, using non-perforated plastic containers (58 cm × 38 cm × 8 cm), each filled with 8 kg of red soil. Plump, undamaged rice seeds were selected, surface-sterilized with 3% H_2_O_2_ for 15 min, rinsed five times with deionized water (DI water), and then soaked in DI water and germinated in the dark at 30 °C in a thermostatic incubator for 24 h. Once seeds showed white tips (radicle length ≤ 2 mm) and uniform sprouting, they were sown using two seedling-raising methods: broadcast sowing and drill sowing, with 60 g of pre-germinated seeds (≈30% moisture content) per container; broadcast sowing involved manual uniform scattering, while drill sowing consisted of sowing 24 even rows longitudinally. At the three-leaf stage, foliar spray treatments were applied at 6:00 PM: seedlings were sprayed with 0.5 mg·L^−1^ GR24 until leaves were thoroughly moistened without dripping, whereas the control group received an equal volume of distilled water. Twenty-four hours after regulator treatment, salt stress was imposed by adding NaCl at 0.4% (*w*/*w*) of soil weight: 32 g of NaCl was dissolved in 2 L of distilled water and slowly poured into each container; the control group received the same volume of distilled water.

Eight treatments were set up in the experiment: D (drill sowing); DN (drill sowing + NaCl); B (broadcast sowing); BN (broadcast sowing + NaCl); DG (drill sowing + GR24); BG (broadcast sowing + GR24); DGN (drill sowing +GR24 + NaCl); and BGN (broadcast sowing + GR24 + NaCl). Each treatment was set with 3 replicates; sampling was conducted on the 3rd, 6th, and 9th days after salt stress, respectively, using the five-point sampling method, where 10 cm^2^ quadrats were randomly selected in non-porous plastic boxes for sampling, and 3 replicate samples were taken from each treatment for the determination of various indicators.

### 2.3. Measurement Items and Methods

#### 2.3.1. Measurement of Morphological Indices

Twenty seedlings were randomly selected. Plant height and root length were measured with a ruler, stem base width with vernier calipers, leaf area with a leaf area meter, and fresh weight with an electronic balance.

#### 2.3.2. Measurement of Leaf Gas Exchange Parameters

Leaf gas exchange parameters of the top-first fully expanded leaf were measured between 9:00 and 11:30 AM on sunny days (with light intensity greater than 1000 μmol·m^−2^·s^−1^) using a portable photosynthesis system (LI-6800, LI-COR, Inc., Lincoln, NE, USA). Parameters included net photosynthetic rate (Pn), stomatal conductance (Gs), intercellular CO_2_ concentration (Ci), and transpiration rate (Tr). Measurements were conducted with an airflow rate of 400 μmol·s^−1^ in the chamber, a carbon dioxide concentration of 400 μmol·mol^−1^, a light intensity set at 1000 μmol·m^−2^·s^−1^, a leaf temperature of 27 ± 1 °C, and a relative air humidity between 65–70%.

#### 2.3.3. Measurement of Chlorophyll Fluorescence Parameters

After sunset, seedlings were moved to a completely dark room for 30 min of dark adaptation. Chlorophyll fluorescence parameters were measured using a Mini-Imaging-PAM modulated chlorophyll fluorescence imaging system (Walz, Effeltrich, Germany), including initial fluorescence (Fo), maximum photochemical efficiency of PSII (Fv/Fm), electron transport rate (ETR), actual quantum yield of PSII (ΦPSII), photochemical quenching coefficient (qP), and non-photochemical quenching coefficient (NPQ).

#### 2.3.4. Observation of Chloroplast Ultrastructure

Following the method of Chaffey [16], rice leaf samples were transversely cut into 2 mm-thick pieces and fixed in 3% glutaraldehyde solution in 0.1 M phosphate buffer (pH 7.4) at 4 °C for 24 h. The samples were then post-fixed in 1% osmium tetroxide solution for 7 h. Subsequently, dehydration was performed using a graded ethanol series (70%, 80%, 90%, and 100%), followed by infiltration with acetone and embedding in Epon 812 epoxy resin. Ultrathin sections were cut using an ultramicrotome (Leica Microsystems GmbH, Wetzlar, Germany; model: Leica EM UC6), stained with uranyl acetate and lead citrate for 15 min each, and observed under a transmission electron microscope (JEOL Ltd., Tokyo, Japan; model: JEOL JEM-1230) for photography.

#### 2.3.5. Determination of Leaf Malondialdehyde (MDA), Hydrogen Peroxide (H_2_O_2_), and Superoxide Anion (O_2_·^−^) Generation Rate

A 0.5 g leaf sample was homogenized in 10 mL of 10% trichloroacetic acid (TCA) and centrifuged at 6000× *g* for 20 min. Malondialdehyde (MDA) content was determined according to the thiobarbituric acid reaction method [17], in which 1 mL of supernatant was added to 2 mL of reaction mixture containing 0.6% (*v*/*v*) thiobarbituric acid (TBA) and 10% (*w*/*v*) TCA. The mixture was boiled at 100 °C for 15 min, cooled, and centrifuged at 4000× *g* for 20 min. The absorbance of the supernatant was measured at 450 nm, 532 nm, and 600 nm. H_2_O_2_ content was determined following Qiu et al. [18] with minor modifications: 0.5 mL supernatant was added to 1 mL of KI solution (e.g., 1 M) and 0.5 mL 10 mM potassium phosphate buffer (pH 7.0). Absorbance was measured at 390 nm, and H_2_O_2_ content was quantified using a standard curve. The O_2_·^−^ production rate was assayed according to the method described by Elstner and Heupel [19]: 0.5 g leaves were homogenized in 5 mL phosphate buffer (50 mM, pH 7.8) in an ice bath and the homogenate was centrifuged at 4 °C and 12,000× *g* for 20 min; then, 0.5 mL of the supernatant, 0.5 mL of phosphate buffer, and 1 mL of 10 mM hydroxylamine hydrochloride were mixed and incubated at 25 °C for 1 h, followed by the addition of 2 mL diethyl ether to extract chlorophyll. Subsequently, 1 mL of 17 mM sulfanilic acid was added first. After incubation at 25 °C for 20 min, 1 mL of 7 mM α-naphthylamine was added, the mixture was centrifuged at 3000× *g* for 3 min, and the absorbance was measured at 530 nm. The O_2_·^−^ production rate was calculated based on a NaNO_2_ standard curve.

#### 2.3.6. Determination of Leaf Relative Electrolyte Conductivity (REC)

Fresh leaf samples (0.1 g) were placed in test tubes containing 10 mL of deionized water. After 24 h, the initial conductivity (E1) was measured using a conductivity meter. The samples were then boiled in a water bath for 20 min, cooled to 25 °C, and the final conductivity (E2) was measured. The conductivity of the deionized water was measured as the blank (E3). The relative electrolyte conductivity (REC) was calculated as follows:

REC = [(E1 − E3)/(E2 − E3)] × 100%(1)

#### 2.3.7. Determination of Leaf Antioxidant Enzyme Activities

Crude enzymes were extracted according to the method described by Lee et al. [20]. A 0.5 g sample of fresh rice leaves was homogenized in 10 mL of phosphate-buffered saline (PBS, pH 7.8), and the homogenate was centrifuged at 10,000 rpm (revolutions per minute) at 4 °C for 15 min to obtain the crude enzyme extract. Superoxide dismutase (SOD) activity was determined using the nitroblue tetrazolium (NBT) method [21]: 100 μL of enzyme extract was added to a mixture containing 14.5 mM methionine, 3 mM EDTA-Na_2_, 60 μM riboflavin, and 2.25 mM nitroblue tetrazolium (NBT); the tubes were placed in an illuminated incubator at 4000 lux and 25 °C for 20 min, and absorbance was measured at 560 nm. Ascorbate peroxidase (APX) activity was assayed following Rahman [22]: 0.1 mL of enzyme extract was added sequentially to the reaction mixture containing 0.1 mmol/L EDTA-Na_2_, 5 mmol/L ascorbic acid (AsA), and 20 mmol/L H_2_O_2_, and absorbance was read at 290 nm. Peroxidase (POD) activity was determined via the oxidation of guaiacol according to Chen et al. [23]: 40 μL of enzyme extract was added to 3 mL of reaction mixture (50 mL PBS (0.2 M, pH 6.0) in a beaker, with 28 μL guaiacol added and stirred while heating on a magnetic stirrer until guaiacol dissolved; after cooling, 19 μL of 30% H_2_O_2_ was added), and absorbance was measured at 470 nm. Catalase (CAT) activity was measured according to Xu et al. [24]: 100 μL of enzyme extract was added to 2.9 mL of reaction mixture (prepared by adding 0.05 mL of 30% H_2_O_2_ to 100 mL PBS (0.15 M, pH 7.0)), and activity was measured as the decrease in absorbance at 240 nm as H_2_O_2_ was consumed.

#### 2.3.8. Determination of Key Enzyme Activities in the ASA-GSH Cycle and Non-Enzymatic Antioxidants

Ascorbic acid (ASA) content was determined according to the method described by Kampfenke et al. [25]: 0.5 g of plant leaves was ground with 10 mL of 5% TCA solution at 4 °C and centrifuged at 20,000 rpm for 15 min; 1.0 mL of the sample extract was pipetted into a test tube; and mixed with 1.0 mL of 5% TCA and 1.0 mL of ethanol; then 0.5 mL of 0.4% H_3_PO_4_ in ethanol, 1.0 mL of 0.5% BP (bathophenanthroline) in ethanol, and 0.5 mL of 0.03% FeCl_3_ in ethanol were added sequentially, making a total volume of 5.0 mL; the solution was incubated at 30 °C for 90 min, and absorbance was measured at 534 nm; and AsA content was calculated using a standard curve. Glutathione (GSH) content was assayed following Tyburski and Tretyn [26]: 0.5 g of plant leaves was ground with 5 mL of 3% TCA solution at 4 °C and centrifuged at 20,000 rpm for 15 min; the supernatant was added sequentially to 150 mmol·L^−1^ NaH_2_PO_4_ (pH 7.7), 6.3 mmol·L^−1^ DTNB (5,5′-dithiobis(2-nitrobenzoic acid)), and 1 mol·L^−1^ NaOH; the solution was incubated at 30 °C for 10 min, and absorbance was measured at 412 nm. Oxidized glutathione (GSSG) content was determined using the DTNB method [27]: 0.5 g of plant leaves was homogenized in 5 mL of ice-cold 5% sulfosalicylic acid at 4 °C and centrifuged at 20,000× *g* for 20 min; the supernatant was added sequentially to 5% sulfosalicylic acid, 1.84 M triethanolamine, and 10% vinylpyridine, and incubated in a water bath at 25 °C for 1 h; then 50 mM PBS (pH 7.5), 10 mM NADPH, and 12.5 mM DTNB were added and incubated at 25 °C for 10 min; finally, 50 U/mL glutathione reductase (GR) was added, and absorbance was read at 412 nm. Frozen leaf samples (0.5 g) were ground into powder in liquid nitrogen, homogenized with 10 mL PBS (50 mmol·L^−1^, pH 7.8), and centrifuged at 12,000× *g* for 20 min at 4 °C. The supernatant was collected as the enzyme extract for assaying GR, DHAR, and MDHAR activities. Glutathione reductase (GR) activity was measured following Zhu et al. [28]: 100 μL enzyme extract was added sequentially to 25 mmol·L^−1^ sodium phosphate buffer (containing 2 mmol·L^−1^ EDTA, pH 7.0), 10 mmol·L^−1^ oxidized glutathione (GSSG), and 24 mmol·L^−1^ NADPH; absorbance was read at 340 nm, and the decrease over 30 s was calculated. Monodehydroascorbate reductase (MDHAR) activity was determined following Shan et al. [29]: the enzyme extract was added sequentially to 25 mmol·L^−1^ sodium phosphate buffer (containing 2 mmol·L^−1^ EDTA, pH 7.0), 7.5 mmol·L^−1^ ascorbic acid (AsA), 2 mmol·L^−1^ NADPH, and ascorbate oxidase (e.g., 10 U/mL); absorbance was measured at 340 nm. Dehydroascorbate reductase (DHAR) activity was assayed according to Hasanuzzaman [30]: 100 μL enzyme extract was added sequentially to 25 mmol·L^−1^ sodium phosphate buffer (containing 2 mmol·L^−1^ EDTA, pH 7.0), 20 mmol·L^−1^ reduced glutathione (GSH), and 10 mmol·L^−1^ dehydroascorbic acid (DHA), with absorbance read at 265 nm.

#### 2.3.9. Determination of Leaf Total Phenolic, Flavonoid Contents, and PAL and PPO Activities

A 0.1 g leaf sample was placed in a 50 mL stoppered conical flask, mixed with 3 mL of 60% ethanol, and ultrasonicated for 40 min. After centrifugation at 2500 rpm for 10 min, the supernatant was transferred to a 50 mL conical flask. The residue was re-extracted three times with 3 mL of 60% ethanol under ultrasonication. The combined supernatants were stored at 4 °C for subsequent analysis. Total phenolic content was determined using the Folin–Ciocalteu method [31]: 0.2 mL extract was mixed with 1.5 mL of 10% Na_2_CO_3_ solution, the mixture was diluted to 10 mL, incubated at 35 °C for 30 min, and absorbance was measured at 765 nm. Flavonoid content was assayed with minor modifications using the NaNO_2_-Al(NO_3_)_3_ colorimetric method [32]: 1.0 mL extract was reacted with 1.0 mL 5% NaNO_2_ for 6 min, followed by 1.0 mL 10% Al(NO_3_)_3_; finally, 4 mL 4% NaOH was added and diluted to 10 mL. After 15 min of reaction, absorbance was measured at 510 nm. Polyphenol oxidase (PPO) activity was determined according to Luh and Phithakpol [33]: 0.5 g leaves were homogenized in cold extraction buffer containing 20 mL of 100 mM sodium phosphate buffer (pH 7.0) and 0.5 g polyvinylpolypyrrolidone (PVPP). The filtrate was centrifuged at 27,000× *g* for 30 min at 4 °C; 1 mL supernatant was mixed with 1 mL sodium phosphate buffer (100 mM, pH 7.0) and 1 mL catechol (50 mM). Absorbance change was spectrophotometrically recorded at 410 nm. Phenylalanine ammonia-lyase (PAL) activity was analyzed following Assis et al. [34] with minor modifications: 0.5 g leaves were homogenized in 50 mM sodium phosphate buffer (pH 8.8) containing 5 mM β-mercaptoethanol. The filtrate was centrifuged at 27,000× *g* for 30 min at 4 °C; 1 mL supernatant was mixed with 2 mL 50 mM borate buffer (pH 8.8) and 1 mL 20 mM L-phenylalanine, and incubated at 37 °C for 120 min. The reaction was terminated with 1 mL 1 M HCl, and absorbance measured at 290 nm.

#### 2.3.10. Determination of Endogenous Hormone Content in Leaves

The content of endogenous hormones was measured by Shanghai Enzyme-linked Biotechnology Co., Ltd. (Shanghai, China). A sandwich enzyme-linked immunosorbent assay (ELISA) kit was used based on a one-step double-antibody sandwich method (Shanghai Enzyme-linked Biotechnology Co., Ltd., Shanghai, China). The absorbance (OD value) was measured using a microplate reader at a wavelength of 450 nm, and the sample concentration was calculated accordingly.

#### 2.3.11. Measurement of Ion Contents in Rice Seedling Leaves

Ion contents (Cl^−^, Ca^2+^, Mg^2+^, Na^+^, K^+^) were determined using inductively coupled plasma mass spectrometry (Agilent Technologies, Santa Clara, CA, USA; model: Agilent 7700 Series ICP-MS) following Kaur et al.’s protocol [35].

### 2.4. Data Analysis

In this study, experimental data were analyzed using one-way analysis of variance (ANOVA) with SPSS 25.0 software (SPSS Inc., Chicago, IL, USA). Multiple comparisons were performed using Duncan’s test, analyzing the mean values and standard errors of three replications, with a significance level of 0.05. Graphs were plotted using Origin 2021 software (OriginLab, Northampton, MA, USA).

## 3. Results

### 3.1. Effects of Sowing Methods and GR24 on Seedling Morphology Under Salt Stress

Under normal conditions, drill-sown seedlings exhibited superior morphological indicators compared to broadcast-sown seedlings. After salt stress, seedlings’ plant height, root length, stem base width, leaf area, and fresh weight significantly decreased. But at 3–9 days of salt stress, the plant height, root length, stem base width, leaf area, and fresh weight of drill-sown seedlings were higher than those of broadcast-sown seedlings. Exogenous spraying of GR24 significantly alleviated the negative effects of salt stress on seedlings. Compared to DN, the DGN treatment significantly increased seedlings’ plant height, root length, stem base width, leaf area, and fresh weight by 16.26–26.98%, 7.00–8.81%, 8.63–10.74%, 16.18–22.98%, and 5.93–8.36%, respectively, at 3–9 days; compared to BN, the BGN treatment significantly increased seedlings’ plant height, root length, stem base width, leaf area, and fresh weight by 7.78–15.56%, 8.23–13.52%, 9.06–13.98%, 2.55–16.46%, and 5.73–8.34%, respectively, at 3–9 days; compared to BGN, the DGN treatment demonstrated superior alleviating effects on morphology to BGN, indicating that spraying GR24 under drill sowing more effectively mitigated salt stress than under broadcast sowing. The results demonstrate that salt stress inhibited rice seedling growth, exogenous GR24 application effectively alleviated this salt stress-induced inhibition, and drill sowing yielded better effects than broadcast sowing (Table 1).

### 3.2. Effects of Sowing Methods and GR24 on Gas Exchange Parameters of Seedlings Under Salt Stress

**Figure 1:**
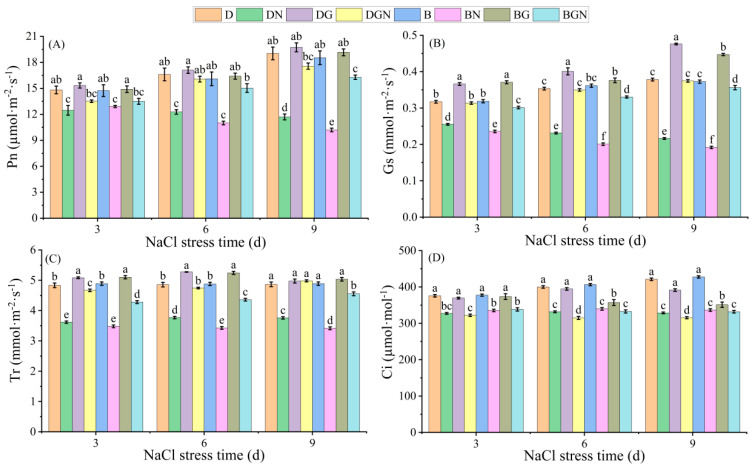
Effects of sowing methods and GR24 on net photosynthetic rate (Pn) (**A**), stomatal conductance (Gs) (**B**), transpiration rate (Tr) (**C**), and intercellular CO_2_ concentration (Ci) (**D**) of seedlings under salt stress. D (drill sowing); DN (drill sowing + NaCl); B (broadcast sowing); BN (broadcast sowing + NaCl); DG (drill sowing + GR24); BG (broadcast sowing + GR24); DGN (drill sowing + GR24 + NaCl); BGN (broadcast sowing + GR24 + NaCl). Data are presented as mean ± standard error of three replicates. Different lowercase letters within a column indicate significant differences among treatments at *p* < 0.05.

Under normal conditions, photosynthesis was stronger in drill-sown seedlings than in broadcast-sown seedlings. After salt stress, seedlings’ Pn, Gs, Tr, and Ci significantly decreased. Exogenous spraying of GR24 altered this situation, improving photosynthetic parameters to varying degrees. Compared to DN, the DGN treatment increased seedlings’ Pn, Gs, Tr, and Ci by 8.43–50.10%, 22.88–73.23%, 29.01–32.58%, and 1.67–4.00%, respectively, at 3–9 days; compared to BN, the BGN treatment increased seedlings’ Pn, Gs, Tr, and Ci by 4.46–59.17%, 27.87–85.83%, 22.98–33.29%, and 0.93–2.07%, respectively, at 3–9 days; compared to BGN, the DGN treatment demonstrated significantly superior improvement effects to BGN, restoring levels approaching non-salt-treated conditions. The results indicate that salt stress limited photosynthetic rate through stomatal restrictions, and GR24 alleviated stomatal limitations, with drill-sown treatments approaching normal levels (Figure 1).

### 3.3. Effects of Sowing Methods and GR24 on Chlorophyll Fluorescence Parameters of Seedlings Under Salt Stress

**Figure 2:**
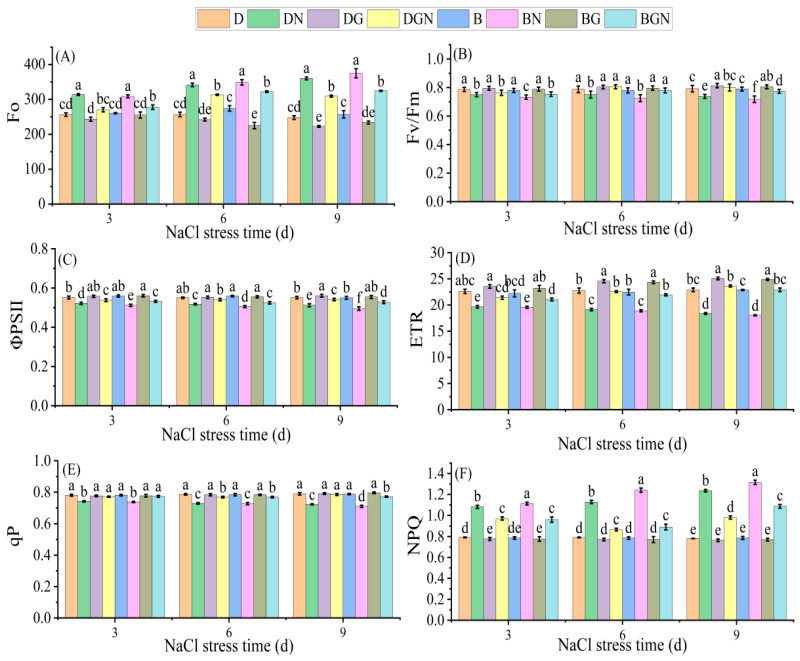
Effects of sowing methods and GR24 on initial fluorescence (Fo) (**A**), maximum photochemical efficiency of PSII (Fv/Fm) (**B**), actual quantum yield of PSII (ΦPSII) (**C**), electron transport rate (ETR) (**D**), photochemical quenching coefficient (qP) (**E**), and non-photochemical quenching coefficient (NPQ) (**F**) of seedlings under salt stress. D (drill sowing); DN (drill sowing + NaCl); B (broadcast sowing); BN (broadcast sowing + NaCl); DG (drill sowing + GR24); BG (broadcast sowing + GR24); DGN (drill sowing + GR24 + NaCl); BGN (broadcast sowing + GR24 + NaCl). Data are presented as mean ± standard error of three replicates. Different lowercase letters within a column indicate significant differences among treatments at *p* < 0.05.

Salt stress damaged photosystem II, leading to continuous decreases in seedlings’ Fv/Fm, ΦPSII, ETR, and qP and significant increases in Fo and NPQ over 3–9 days under salt stress. Exogenous spraying of GR24 significantly improved chlorophyll fluorescence parameters in salt-treated seedlings. Compared to DN, DGN treatment increased seedlings’ Fv/Fm, ΦPSII, ETR, and qP by 1.73–8.45%, 3.07–5.73%, 8.80–28.68%, and 3.91–8.62%, respectively, while decreasing Fo and NPQ by 8.29–14.15% and 10.23–20.67%, respectively, at 3–9 days; compared to BN, BGN treatment increased seedlings’ Fv/Fm, ΦPSII, ETR, and qP by 2.82–7.76%, 3.83–6.60%, 7.67–26.99%, and 4.83–8.75%, respectively, while decreasing Fo and NPQ by 7.64–13.42% and 13.61–28.23%, respectively, at 3–9 days; DGN treatment restored Fv/Fm, ETR, and qP to non-salt-treated levels by day 9. The results demonstrate that salt stress impaired photosystem II, and spraying GR24 under drill sowing restored it to near-normal levels (Figure 2).

### 3.4. Effects of Sowing Methods and GR24 on Chloroplast Ultrastructure of Seedlings Under Salt Stress

**Figure 3:**
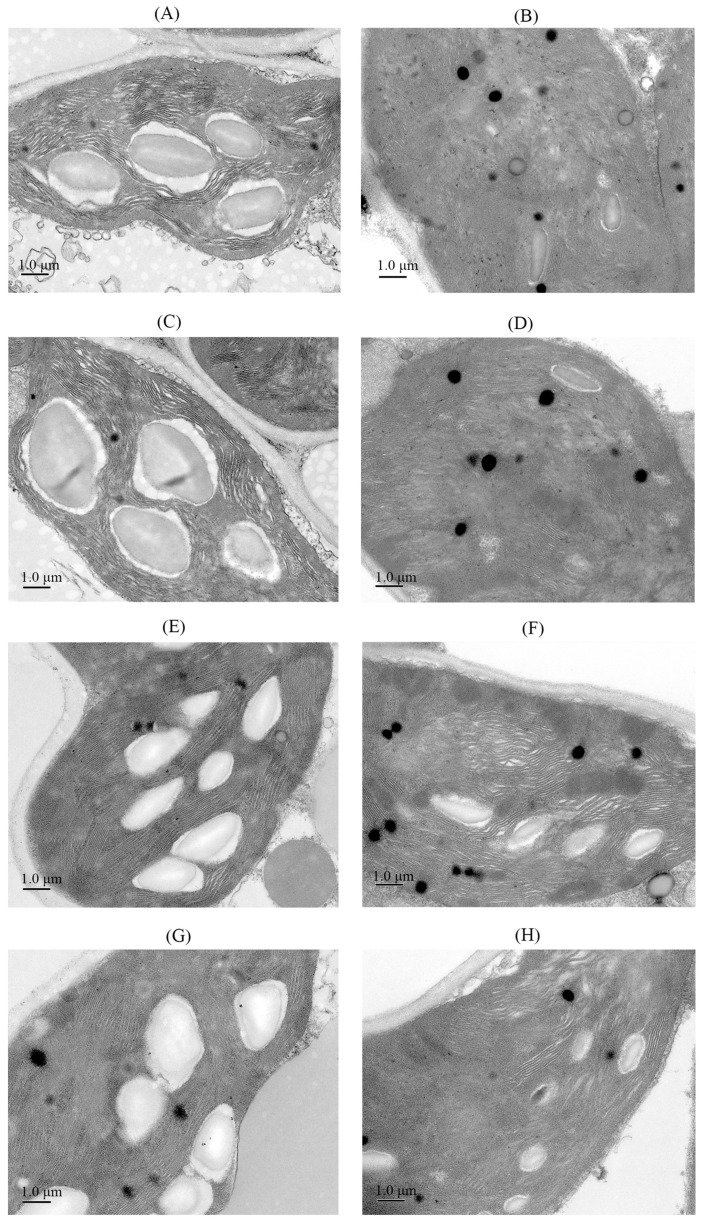
Effects of sowing method and GR24 on chloroplast ultrastructure of seedlings under salt stress. (**A**): drill sowing; (**B**): drill sowing + NaCl; (**C**): broadcast sowing; (**D**): broadcast sowing + NaCl; (**E**): drill sowing + GR24; (**F**): drill sowing + GR24 + NaCl; (**G**): broadcast sowing + GR24; (**H**): broadcast sowing + GR24 + NaCl.

As shown in Figure 3, under non-stress conditions, drill-sown and broadcast-sown rice seedlings exhibited neatly arranged thylakoid lamellae, clearly defined oval starch grains, and few osmiophilic granules (Figure 3A,C). After salt stress, chloroplasts showed severe swelling, structural disorganization, disintegration of thylakoid membranes and grana, decomposition of starch grains, and increased osmiophilic granules compared to non-salt treatments (Figure 3B,D). Exogenous GR24 application increased the number of starch grains in chloroplasts of non-stressed seedlings (Figure 3E,G). Under salt stress, GR24 alleviated chloroplast swelling, promoted more ordered thylakoids and grana, and increased starch grain content (Figure 3F,H), with drill-sown seedlings exhibiting higher starch grain accumulation. The results indicate that salt stress damaged the chloroplast structure, causing the disintegration of thylakoid membranes and grana, and that spraying GR24 can repair the chloroplast structure.

### 3.5. Effects of Sowing Methods and GR24 on Membrane Damage in Seedlings Under Salt Stress

**Figure 4:**
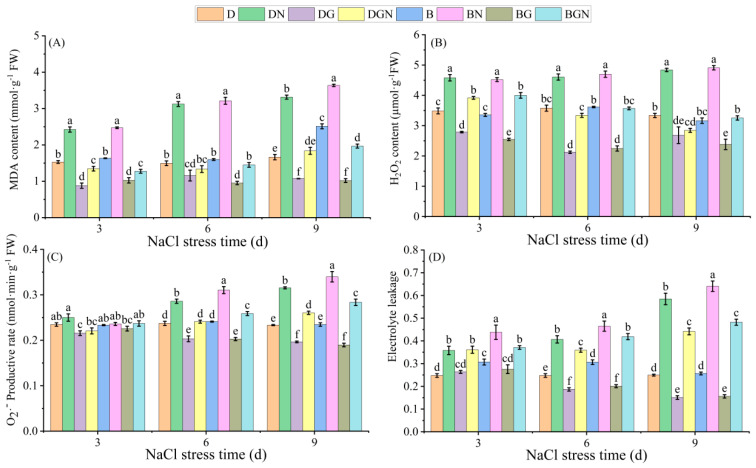
Effects of sowing methods and GR24 on malondialdehyde content (MDA) (**A**), hydrogen peroxide (H_2_O_2_) content (**B**), superoxide anion (O_2_·^−^) production rate (**C**), and electrolyte leakage (**D**) of seedlings under salt stress. D (drill sowing); DN (drill sowing + NaCl); B (broadcast sowing); BN (broadcast sowing + NaCl); DG (drill sowing + GR24); BG (broadcast sowing + GR24); DGN (drill sowing + GR24 + NaCl); BGN (broadcast sowing + GR24 + NaCl). Data are presented as mean ± standard error of three replicates. Different lowercase letters within a column indicate significant differences among treatments at *p* < 0.05.

Salt stress over 3–9 days continuously increased leaf MDA, H_2_O_2_, O_2_·^−^ content, and electrolyte leakage in seedlings. Exogenous spraying of GR24 reversed this trend, significantly reducing seedlings’ MDA, H_2_O_2_, O_2_·^−^ content, and electrolyte leakage. Compared to DN, DGN treatment decreased leaf MDA, H_2_O_2_, O_2_·^−^ content, and electrolyte leakage by 44.48–57.29%, 14.47–41.19%, 11.76–17.44%, and 0.86–24.45%, respectively, at 3–9 days; compared to BN, BGN treatment decreased MDA, H_2_O_2_, O_2_·^−^ content, and electrolyte leakage by 45.89–54.84%, 11.49–33.71%, 0.43–16.54%, and 15.30–24.72%, respectively, at 3–9 days, with DGN exhibiting lower membrane lipid peroxide content and electrolyte leakage than BGN. The results indicate that salt stress caused substantial accumulation of membrane lipid peroxides in seedlings, while spraying GR24 reduced their accumulation (Figure 4).

### 3.6. Effects of Sowing Methods and GR24 on Antioxidant Enzyme Activities in Seedling Leaves Under Salt Stress

**Figure 5:**
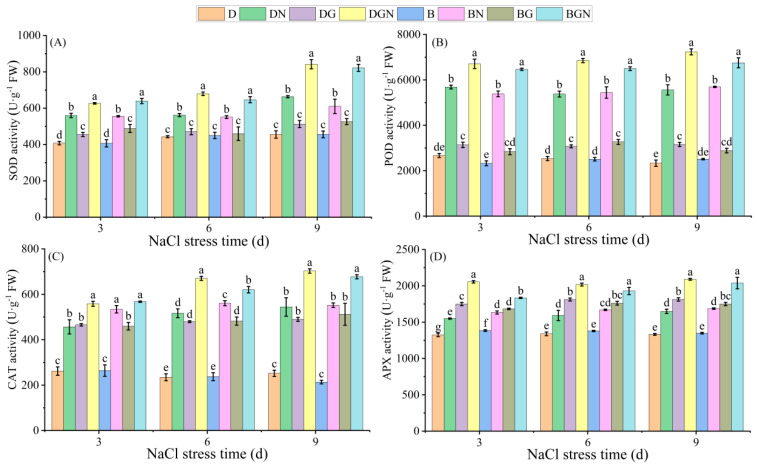
Effects of sowing methods and GR24 on superoxide dismutase activity (SOD) (**A**), peroxidase activity (POD) (**B**), catalase activity (CAT) (**C**), and ascorbate peroxidase activity (APX) (**D**) of seedlings under salt stress. D (drill sowing); DN (drill sowing + NaCl); B (broadcast sowing); BN (broadcast sowing + NaCl); DG (drill sowing + GR24); BG (broadcast sowing + GR24); DGN (drill sowing + GR24 + NaCl); BGN (broadcast sowing + GR24 + NaCl). Data are presented as mean ± standard error of three replicates. Different lowercase letters within a column indicate significant differences among treatments at *p* < 0.05.

Antioxidant enzyme activities (POD, SOD, APX, CAT) in seedling leaves increased significantly over 3–9 days after salt stress. DN and BN treatments differentially affected the activities of the same antioxidant enzymes at identical time points. Exogenous GR24 further enhanced antioxidant enzyme activities. Compared to DN, DGN treatment significantly increased leaf SOD, POD, CAT, and APX activities by 11.91–26.94%, 17.95–30.17%, 22.33–29.09%, and 26.77–32.63%, respectively, at 3–9 days; compared to BN, BGN treatment significantly increased leaf SOD, POD, CAT, and APX activities by 15.18–34.67%, 18.60–20.13%, 6.29–22.66%, and 12.35–20.81%, respectively, at 3–9 days. DGN demonstrated overall superior enhancement of antioxidant enzyme activities compared to BGN. The results indicate that seedlings elevated antioxidant enzyme activities to defend against salt stress, while GR24 further boosted these activities to combat salt stress, with drill sowing yielding better enhancement effects (Figure 5).

### 3.7. Effects of Sowing Methods and Exogenous GR24 on Key AsA-GSH Cycle Enzyme Activities and Non-Enzymatic Antioxidants in Seedling Leaves Under Salt Stress

**Figure 6:**
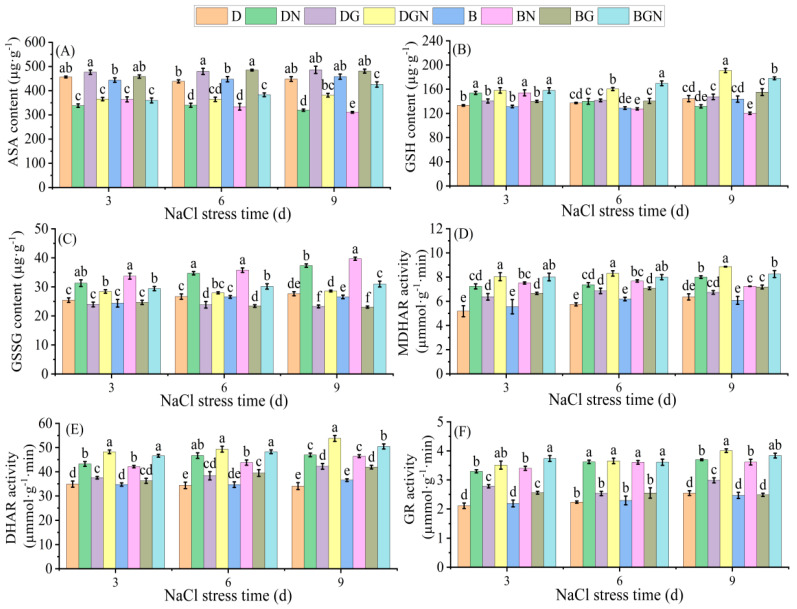
Effects of sowing methods and GR24 on ascorbic acid (AsA) content (**A**), reduced glutathione (GSH) content (**B**), oxidized glutathione (GSSG) content (**C**), monodehydroascorbate reductase (MDHAR) activity (**D**), dehydroascorbate reductase (DHAR) activity (**E**), and glutathione reductase (GR) activity (**F**) of seedlings under salt stress. D (drill sowing); DN (drill sowing + NaCl); B (broadcast sowing); BN (broadcast sowing + NaCl); DG (drill sowing + GR24); BG (broadcast sowing + GR24); DGN (drill sowing + GR24 + NaCl); BGN (broadcast sowing + GR24 + NaCl). Data are presented as mean ± standard error of three replicates. Different lowercase letters within a column indicate significant differences among treatments at *p* < 0.05.

Under normal conditions, key ASA-GSH cycle enzyme activities and non-enzymatic antioxidants in seedlings’ leaves showed no significant differences between sowing methods. After salt stress (3–9 days), leaf ASA and GSH contents continuously decreased significantly, while GSSG content and MDHAR, DHAR, and GR activities continuously increased significantly. DN treatment exhibited higher ASA and GSH contents and higher MDHAR, DHAR, GR activities than BN. Exogenous GR24 differentially enhanced ASA-GSH substances and enzyme activities. Compared to DN, DGN treatment significantly increased ASA content, GSH content, and MDHAR, DHAR, and GR activities by 7.93–19.31%, 2.71–44.97%, 10.76–12.78%, 5.62–14.51%, and 0.87–8.39%, respectively, while significantly decreasing GSSG content by 9.14–23.43% at 3–9 days; compared to BN, BGN treatment significantly increased ASA content, GSH content, and MDHAR, DHAR, and GR activities by 14.80–37.04%, 2.60–48.24%, 4.16–13.99%, 8.67–10.68%, and 0.09–10.00%, respectively, while significantly decreasing GSSG content by 12.67–21.95% at 3–9 days. DGN treatment demonstrated superior improvement in ASA-GSH substances and enzyme activities compared to BGN. The results indicate that GR24 increased antioxidant content and enhanced related enzyme activities, with better effects under drill sowing than broadcast sowing (Figure 6).

### 3.8. Effects of Sowing Methods and GR24 on Total Phenolics, Flavonoids, and Related Enzyme Activities in Seedling Leaves Under Salt Stress

**Figure 7:**
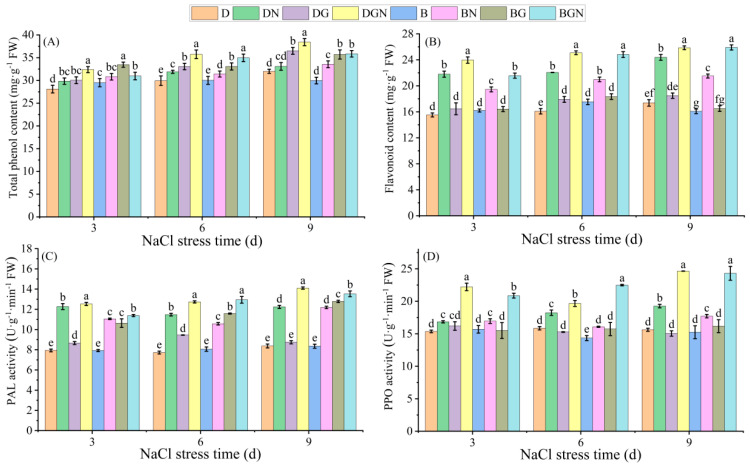
Effects of sowing methods and GR24 on total phenolics content (**A**), flavonoids content (**B**), phenylalanine ammonia-lyase (PAL) activity (**C**), and polyphenol oxidase (PPO) activity (**D**) of seedlings under salt stress. D (drill sowing); DN (drill sowing + NaCl); B (broadcast sowing); BN (broadcast sowing + NaCl); DG (drill sowing + GR24); BG (broadcast sowing + GR24); DGN (drill sowing + GR24 + NaCl); BGN (broadcast sowing + GR24 + NaCl). Data are presented as mean ± standard error of three replicates. Different lowercase letters within a column indicate significant differences among treatments at *p* < 0.05.

Under normal conditions, seedlings’ leaf total phenolic and flavonoid contents and PAL and PPO activities showed no significant differences between sowing methods. After salt stress, leaf total phenolic and flavonoid contents and PAL and PPO activities significantly increased. Exogenous GR24 further enhanced total phenolic and flavonoid contents and PAL and PPO activities. Compared to DN, DGN treatment significantly increased seedlings’ leaf total phenolics, flavonoids, PAL activity, and PPO activity by 8.47–16.11%, 6.01–9.94%, 2.12–15.20%, and 7.82–31.94%, respectively, at 3–9 days; compared to BN, BGN treatment significantly increased total phenolics, flavonoids, PAL activity, and PPO activity by 5.03–11.34%, 10.86–20.45%, 2.99–22.42%, and 23.13–39.92%, respectively, at 3–9 days. DGN exhibited significantly higher total phenolic content and PAL activity than BGN. The results indicate that GR24 improved seedling salt tolerance by enhancing total phenolic/flavonoid contents and PAL/PPO activities, with superior effects under drill sowing (Figure 7).

### 3.9. Effects of Sowing Methods and GR24 on Endogenous Hormone Content in Seedlings Under Salt Stress

**Figure 8:**
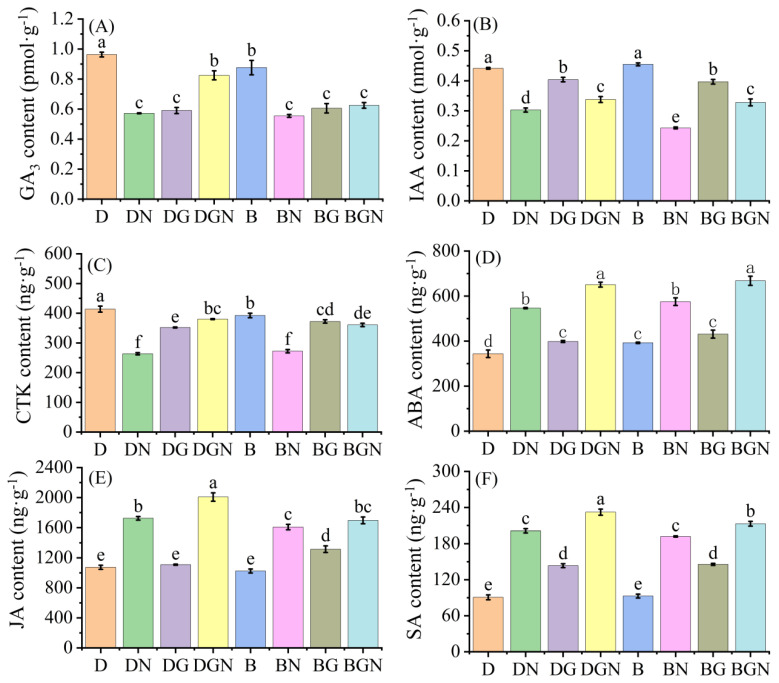
Effects of sowing methods and GR24 on gibberellin A3 (GA3) (**A**), auxin (IAA) (**B**), cytokinin (CTK) (**C**), abscisic acid (ABA) (**D**), jasmonic acid (JA) (**E**), and salicylic acid (SA) (**F**) of seedlings under salt stress. D (drill sowing); DN (drill sowing + NaCl); B (broadcast sowing); BN (broadcast sowing + NaCl); DG (drill sowing + GR24); BG (broadcast sowing + GR24); DGN (drill sowing + GR24 + NaCl); BGN (broadcast sowing + GR24 + NaCl). Data are presented as mean ± standard error of three replicates. Different lowercase letters within a column indicate significant differences among treatments at *p* < 0.05.

Under normal conditions, endogenous hormone contents in seedlings’ leaves showed no significant differences between sowing methods. After salt stress, leaf GA_3_, IAA, and CTK contents significantly decreased, while ABA, JA, and SA contents significantly increased. Exogenous GR24 reduced GA_3_, IAA, and CTK contents in non-salt-stressed seedlings but increased them under salt stress. Compared to DN, DGN treatment significantly increased seedlings’ leaf GA_3_, IAA, CTK, ABA, JA, and SA contents by 44.27%, 11.59%, 44.22%, 19.10%, 16.45%, and 15.33%, respectively, at day 9; compared to BN, BGN treatment significantly increased GA_3_, IAA, CTK, ABA, JA, and SA contents by 12.64%, 34.97%, 32.44%, 16.15%, 5.56%, and 10.83%, respectively, at day 9. DGN exhibited higher endogenous hormone contents than BGN. The results indicate that exogenous GR24 maintains endogenous hormone balance in rice seedlings under salt stress. (Figure 8).

### 3.10. Effects of Sowing Methods and GR24 on Ion Content in Seedlings Under Salt Stress

**Figure 9:**
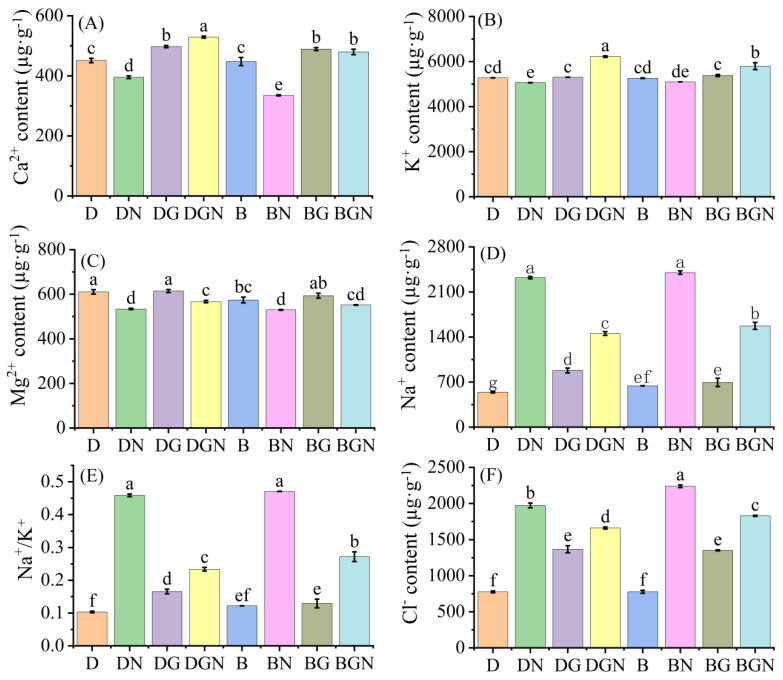
Effects of sowing methods and GR24 on Ca^2+^ content (**A**), K^+^ content (**B**), Mg^2+^ content (**C**), Na^+^ content (**D**), Na^+^/K^+^ ratio (**E**), and Cl^−^ content (**F**) of seedlings under salt stress. D (drill sowing); DN (drill sowing + NaCl); B (broadcast sowing); BN (broadcast sowing + NaCl); DG (drill sowing + GR24); BG (broadcast sowing + GR24); DGN (drill sowing +GR24 + NaCl); BGN (broadcast sowing + GR24 + NaCl). Data are presented as mean ± standard error of three replicates. Different lowercase letters within a column indicate significant differences among treatments at *p* < 0.05.

Under normal conditions, drill-sown seedlings had higher leaf Mg^2+^ and Ca^2+^ contents than broadcast-sown seedlings. At day 9 after salt stress, seedlings’ leaf Cl^−^, K^+^, and Na^+^ contents and Na^+^/K^+^ ratio significantly increased, while Ca^2+^ and Mg^2+^ contents significantly decreased; compared to BN, DN treatment had higher Ca^2+^, Mg^2+^, and K^+^ contents but lower Cl^−^ and Na^+^ contents and Na^+^/K^+^ ratio than BN. Exogenous GR24 increased Ca^2+^, Mg^2+^, and K^+^ contents while reducing Cl^−^ and Na^+^ accumulation, thereby lowering Na^+^/K^+^ ratio. Compared to DN, DGN treatment significantly increased leaf Ca^2+^, Mg^2+^, and K^+^ contents by 33.91%, 6.21%, and 22.82%, respectively, while significantly decreasing Cl^−^ content, Na^+^ content, and Na^+^/K^+^ ratio by 15.81%, 37.38%, and 49.01%, respectively, at day 9; compared to BN, BGN treatment significantly increased leaf Ca^2+^, Mg^2+^, and K^+^ contents by 43.14%, 4.16%, and 13.66%, respectively, while significantly decreasing Cl^−^ content, Na^+^ content, and Na^+^/K^+^ ratio by 18.27%, 34.45%, and 42.19%, respectively, at day 9; compared to BGN, DGN treatment had higher leaf Ca^2+^, Mg^2+^, and K^+^ contents but lower Cl^−^ content, Na^+^ content, and Na^+^/K^+^ ratio than BGN at day 9. The results indicate that under normal conditions, drill-sown seedlings accumulated more Mg^2+^ and Ca^2+^ than broadcast-sown seedlings; additionally, salt stress disrupted ion homeostasis in seedlings, while GR24 restored ion balance and reduced ionic toxicity, with superior effects under drill sowing (Figure 9).

### 3.11. Correlation Between Various Indicators

**Figure 10:**
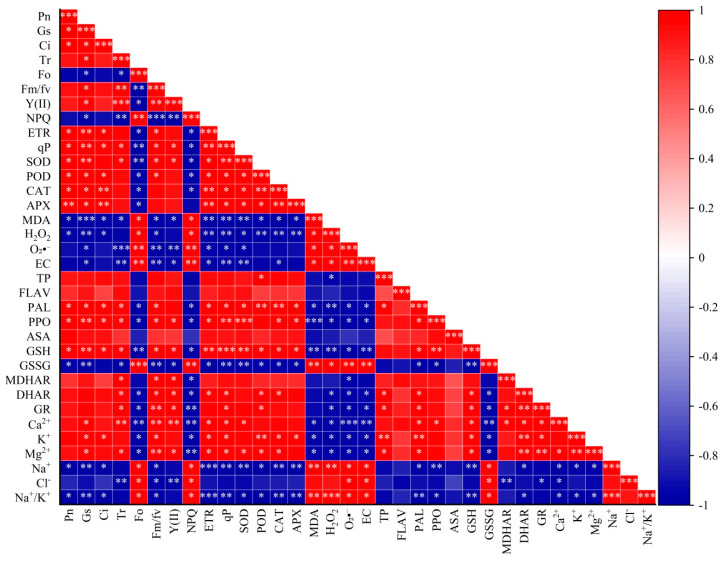
***, ** and * indicate the correlation was extremely significant and significant at 0.001, 0.01 and 0.05 level, respectively. Net photosynthetic rate (Pn), stomatal conductance (Gs), transpiration rate (Tr), intercellular CO_2_ concentration (Ci), initial fluorescence (Fo), maximum photochemical efficiency of PSII (Fv/Fm) actual quantum yield of PSII (ΦPSII), electron transport rate (ETR), photochemical quenching coefficient (qP), non-photochemical quenching coefficient, malondialdehyde content (MDA), hydrogen peroxide (H_2_O_2_), superoxide anion (O_2_·^−^), electrolyte leakage (D), superoxide dismutase (SOD), peroxidase (POD), catalase (CAT), ascorbate peroxidase (APX) (D), ascorbic acid (AsA), reduced glutathione (GSH), oxidized glutathione (GSSG), monodehydroascorbate reductase (MDHAR), dehydroascorbate reductase (DHAR), glutathione reductase (GR), total phenolics (TP), flavonoids (FLAV), phenylalanine ammonia-lyase (PAL), and polyphenol oxidase (PPO).

The results indicated that rice gas exchange parameters (Pn, Gs, Tr, Ci) and chlorophyll fluorescence parameters (Fv/Fm, ΦPSII, ETR, qP) showed significant or highly significant positive correlations with antioxidant enzymes (SOD, POD, CAT, APX), GSH, Ca^2+^, K^+^, and Mg^2+^, while exhibiting significant or highly significant negative correlations with MDA, H_2_O_2_, O_2_·^−^, electrolyte leakage, Na^+^, and the Na^+^/K^+^ ratio. Antioxidant enzymes (SOD, POD, CAT, APX), non-enzymatic antioxidants (GSH, total phenolics), and related enzymes (MDHAR, DHAR, GR, PAL, PPO) showed significant or highly significant positive correlations with Ca^2+^, K^+^, and Mg^2+^, while demonstrating significant or highly significant negative correlations with MDA, H_2_O_2_, O_2_·^−^, electrolyte leakage, Na^+^, and the Na^+^/K^+^ ratio (Figure 10).

## 4. Discussion

### 4.1. Alleviating Effects of Sowing Method and GR24 on Seedling Growth Under Salt Stress

Drill-sown rice seedlings exhibit better ventilation and light transmittance than broadcast-sown seedlings, facilitating seedling growth [36]. This study confirms that drill sowing promotes superior morphological development in rice seedlings under normal growth conditions compared to broadcast sowing. However, prolonged salt stress inhibits rice seedling growth, with plant height, root length, stem base width, leaf area, and fresh weight showing declining trends—consistent with findings in wheat [37] and sorghum [38] under salt stress. Exogenous GR24 application not only promotes seedling growth under normal conditions but also exerts significant protective effects in saline environments, markedly alleviating reductions in plant height, root length, stem base width, leaf area, and fresh weight. This aligns with experimental results in tomatoes [39], fully demonstrating GR24’s efficacy in activating plant salt-tolerance mechanisms and reversing salt stress-induced growth inhibition. Notably, under salt stress, drill-sown seedlings demonstrate superior stress resistance, with all morphological indicators surpassing those of broadcast-sown seedlings under salt stress. The significantly better morphological improvement from GR24 application under drill sowing versus broadcast sowing is attributed to more uniform seedling distribution in drill-sown systems.

### 4.2. Alleviating Effects of Sowing Method and GR24 on the Photosynthetic System of Seedlings Under Salt Stress

Photosynthesis is the energy source and material source for plant survival, and maintaining plant photosynthesis is an important mechanism for enhancing plant salt tolerance [40]. Salt stress generally affects plant Pn through stomatal or non-stomatal factors [41]. In this study, drill-sown seedlings exhibited superior Pn to broadcast-sown seedlings under normal conditions. Salt stress significantly decreased seedlings’ Gs, Tr, and Ci, imposing stomatal limitation on Pn. Exogenous GR24 application restored Ci, Gs, and Tr to near non-stress levels, alleviating stomatal limitation and markedly improving Pn. This likely occurs through GR24 activating the strigolactone signaling pathway to optimize stomatal movement and mesophyll cell photosynthetic activity [42], which aligns with cucumber findings under salt stress [43]. The mitigation effect under drill sowing exceeded broadcast sowing, indicating optimized plant spatial arrangement in drill sowing potentially enhances water and CO_2_ diffusion efficiency [44]. Chlorophyll fluorescence parameters reflect PSII damage and repair [45]. Our results showed that salt stress caused continuous declines in Fv/Fm, ΦPSII, ETR, and qP alongside significant increases in Fo and NPQ, consistent with the stress response pattern featuring PSII reaction center damage (elevated Fo), reduced light capture efficiency (decreased Fv/Fm), and excess energy dissipation as heat (elevated NPQ) [46]. Under GR24 treatment, seedlings exhibited significantly reduced Fo and NPQ alongside elevated Fv/Fm, ΦPSII, ETR, and qP. Exogenous GR24 repairs reaction centers and enhances electron transport chain activity and photochemical efficiency, thereby significantly improving PSII functionality under salt stress, with similar findings reported in rapeseed [12]. Drill-sown seedlings restored Fv/Fm, ETR, and qP to near non-stressed control levels, significantly exceeding recovery in broadcast sowing. This corroborates gas exchange parameter recovery, revealing physiological synergy between drill sowing and GR24: drill sowing establishes a stress-resistant morphological framework while GR24 regulates stomatal function and improves light energy capture efficiency.

### 4.3. Sowing Methods and GR24 Reduce Oxidative Damage in Seedlings by Enhancing Antioxidant Enzyme Activity and Non-Enzymatic Antioxidant Contents

Salt stress-induced accumulation of reactive oxygen species (ROS) is a major factor causing damage to rice seedlings [47]. Malondialdehyde (MDA), a key indicator of membrane lipid peroxidation, reflects the degree of membrane damage under stress [48]. Under salt stress, the plant antioxidant system scavenges excess ROS [49]. In this study, continuous increases in seedlings’ leaf MDA, H_2_O_2_, O_2_·^−^ content, and electrolyte leakage occurred in salt-stressed seedlings, while the antioxidant enzyme system (POD, SOD, APX, CAT) exhibited significantly increased activities. However, their scavenging efficiency remained insufficient to counteract persistent ROS generation, leading to chloroplast structural damage, disintegration of thylakoid membranes and grana, and increased osmiophilic granules—consistent with previous findings [50]. Exogenous GR24 significantly enhanced antioxidant enzyme activities, reduced MDA and ROS levels, maintained biomembrane integrity, and lowered electrolyte leakage, aligning with studies by Ma et al. [51] and Faisal et al. [52]. GR24-treated chloroplasts showed increased starch granules and repaired thylakoid membranes and grana, thereby significantly improving chlorophyll fluorescence parameters. Notably, under salt stress, drill sowing combined with GR24 application exhibited higher antioxidant enzyme activities, reduced ROS and MDA accumulation, and better maintained chloroplast structural stability than broadcast sowing.

The antioxidant defense mechanism in higher plants comprises enzymatic and non-enzymatic components [53]. Ascorbate (AsA) and glutathione (GSH) are primary non-enzymatic antioxidants. The AsA-GSH cycle effectively scavenges metabolically generated H_2_O_2_, playing a critical role in ROS elimination [54]. This study found that post-stress seedlings’ leaf AsA and GSH contents significantly decreased, while oxidized glutathione (GSSG) content and key enzyme (MDHAR, DHAR, GR) activities significantly increased. Exogenous GR24 altered this response by elevating AsA and GSH contents while coordinately activating MDHAR, DHAR, and GR activities, thereby enhancing plant antioxidant capacity—consistent with findings in salt-stressed wheat [55]. This reflects an adaptive strategy where plants upregulate the AsA-GSH cycle to maintain cellular redox homeostasis [56]. Drill-sown seedlings under salt stress exhibited significantly superior improvement to broadcast-sown seedlings. GR24-induced GR activity showed greater enhancement in drill-sown seedlings, more efficiently catalyzing GSSG reduction to GSH and ensuring sufficient GSH pool capacity. Elevated GSH levels directly supported DHAR activity in reducing dehydroascorbate (DHA) to AsA [57]. The significant increase in AsA content in drill-sown seedlings synergized with enhanced APX activity, jointly scavenging H_2_O_2_. This explains why GR24 application under salt stress minimized membrane lipid peroxidation and chloroplast damage most effectively in drill-sown seedlings.

Flavonoids are important polyphenolic compounds and antioxidant secondary metabolites in higher plants [58]. Their presence promotes peroxidase activity and reduces MDA and ROS content [59]. In this study, salt stress significantly increased total phenolic and flavonoid contents alongside key enzyme (PAL, PPO) activities in seedlings’ leaves. Exogenous GR24 further amplified this stress response, significantly elevating total phenolic and flavonoid contents while coordinately activating PAL and PPO activities—consistent with previous research [60]—thereby strengthening the chemical defense system of rice seedlings through activation of the phenylpropanoid pathway. The increase under drill sowing slightly exceeded that under broadcast sowing, presumably attributable to superior photosynthesis in drill sowing providing ample substrates and energy for phenolic compound synthesis.

### 4.4. Sowing Methods and GR24 Alleviate Salt Stress by Regulating Hormone Content in Seedlings

Plant physiological processes are often regulated not by a single hormone but through synergistic and antagonistic interactions among multiple hormones that collectively influence growth and development [61]. Abscisic acid (ABA) serves as a stress hormone playing a critical role in plant abiotic stress tolerance [62], while hormones like gibberellic acid (GA_3_) also significantly contribute to stress resistance [63]. This study found that during the post-stress period, growth-promoting hormones (GA_3_, IAA, CTK) significantly decreased, whereas stress-signaling hormones (ABA, JA, SA) sharply increased—aligning with prior observations that salt stress reduces GA_3_ and IAA while increasing ABA in maize [64]. Exogenous GR24 application substantially elevated GA_3_, IAA, CTK, ABA, JA, and SA contents, indicating its capacity to modulate endogenous hormone homeostasis in rice. This hormonal rebalancing facilitates intracellular ROS scavenging and activates defense systems, thereby enhancing salt tolerance—consistent with established research outcomes [65].

### 4.5. Sowing Methods and GR24 Alleviate Salt Stress by Balancing Ion Content in Seedlings

In plant physiological responses to adversity, homeostasis of mineral elements plays a crucial role. K^+^, as one of the most abundant cations in plant cells, primarily participates in cellular osmoregulation [66]. Ca^2+^ functions as a signaling molecule central to stress signal transduction pathways [67]. Mg^2+^, being the core element for chlorophyll synthesis, directly affects chlorophyll synthesis efficiency and photosynthetic system integrity [68]. Salt stress disrupts plant cellular homeostasis by inducing ionic toxicity and nutritional imbalance [69]. This study found that salt stress caused ion dysregulation in seedlings: Cl^−^, Na^+^, and Na^+^/K^+^ ratio surged, while K^+^, Ca^2+^, and Mg^2+^ contents decreased, indicating competitive inhibition of other ions’ uptake channels by salt ions. Exogenous GR24 application reinforced ion homeostasis, increasing Ca^2+^, Mg^2+^, and K^+^ contents while suppressing Cl^−^ and Na^+^ accumulation and reducing Na^+^/K^+^ ratio, aligning with findings in maize [70]. Notably, drill-sown treatments exhibited higher Ca^2+^, Mg^2+^, and K^+^ contents alongside lower Cl^−^, Na^+^, and Na^+^/K^+^ ratio than broadcast sowing. This demonstrates synergistic advantages between drill sowing and GR24: drill sowing establishes uniformly distributed root architecture, while GR24 enhances ion transport [71], collectively maintaining ion homeostasis under salt stress.

### 4.6. Correlation Analysis Between Various Indicators of Rice Seedlings

This study observed positive correlations between gas exchange parameters (Pn, Gs, Tr, Ci) and chlorophyll fluorescence parameters (Fv/Fm, ΦPSII, ETR, qP) with antioxidant enzymes (SOD, POD, CAT, APX), GSH, and ions (Ca^2+^, K^+^, Mg^2+^), indicating that under salt stress, the antioxidant system and ion homeostasis jointly protect photosynthetic function. Optimization of the sowing method (drill sowing) combined with GR24-enhanced ROS scavenging capacity alleviates oxidative damage to thylakoid membranes and PSII reaction centers, thereby maintaining light energy conversion efficiency (ΦPSII, ETR), aligning with previous research [72]. Negative correlations between the antioxidant system and oxidative damage indicators (MDA, H_2_O_2_, O_2_·^−^, electrolyte leakage) as well as Na^+^ content and Na^+^/K^+^ ratio demonstrate the critical roles of ROS scavenging and ion balance in salt tolerance. Salt stress-induced Na^+^ accumulation disrupts cellular osmotic balance and enzyme structures, triggering excessive ROS production that damages membrane integrity (increased electrolyte leakage) and impairs photosynthetic machinery [73]. GR24 application—particularly under drill sowing—effectively reduces Na^+^ accumulation and ROS levels, thereby mitigating oxidative stress and its inhibition of photosynthesis, a mechanism consistent with existing studies [74]. Drill sowing’s uniform plant arrangement enhances light utilization efficiency, gas diffusion efficiency, and root–soil contact efficiency [75]. This structural advantage synergizes with GR24’s physiological regulation, more effectively protecting rice seedlings under salt stress.

## 5. Conclusions

The results of this study indicate that under normal conditions, the quality of drill-sown seedlings is superior to that of broadcast-sown seedlings. Salt stress inhibits rice growth and development by disrupting the photosynthetic system and hormonal balance of rice seedlings through ionic toxicity and peroxidative damage. Exogenous spraying of GR24 scavenges excess ROS and MDA by increasing antioxidant enzyme activities and non-enzymatic antioxidant contents, maintains ion and hormonal balance, and repairs the photosynthetic system. Due to their better spatial distribution, drill-sown seedlings synergize with GR24 to alleviate the inhibition of salt stress on seedling growth, exhibiting better salt tolerance than broadcast-sown seedlings. However, potential limitations need to be considered for the large-scale field application of the results of this study: the economic cost of GR24 may restrict its popularization in large-area farmland; meanwhile, GR24 is easily degraded by factors such as light, temperature, and precipitation in the natural environment, which may reduce the stability and persistence of field application. Future research can bridge laboratory and field applications: first, conduct field validation trials of different concentrations of GR24, and optimize the application dosage and frequency combined with cost–benefit analysis to reduce application costs; second, develop combinations with biological carriers (such as microbial agents) to improve their stability in the natural environment; and third, explore the synergistic effects of GR24 with other agronomic practices (such as water-saving irrigation and saline–alkali soil amendments) to enhance the comprehensive salt tolerance regulation effect. These efforts will help promote the transformation of the conclusions of this study from laboratory validation to practical field application, providing more operable technical references for rice production in saline–alkali soils.

## Figures and Tables

**Table 1:** Effects of sowing methods and GR24 on plant height, root length, stem base width, leaf area, and fresh weight of rice seedlings under salt stress.

Salinity Sampling Time (d)	Treatments	Pattern Indicator				
		Plant Height (cm)	Length of Root (cm)	Stem Diameter (mm)	Leaf Area (cm^2^)	Fresh Weight (×10^−2^ g)
	D	18.57 ± 0.36 ab	9.16 ± 0.28 a	1.90 ± 0.02 cd	462.23 ± 9.44 cd	19.64 ± 0.40 bc
	DN	15.11 ± 0.46 c	8.19 ± 0.17 cd	1.75 ± 0.03 e	409.35 ± 8.37 de	17.61 ± 0.12 d
	DG	19.71 ± 0.57 a	9.08 ± 0.22 ab	2.10 ± 0.02 a	567.17 ± 27.51 a	21.10 ± 0.30 a
3	DGN	17.57 ± 0.25 b	8.76 ± 0.04 ab	1.93 ± 0.04 cd	503.42 ± 20.21 bc	18.91 ± 0.30 c
	B	18.22 ± 0.39 b	8.82 ± 0.06 ab	1.86 ± 0.03 d	447.12 ± 7.41 cde	19.53 ± 0.10 c
	BN	14.86 ± 0.38 c	7.77 ± 0.14 d	1.73 ± 0.02 d	395.01 ± 11.87 e	16.21 ± 0.35 e
	BG	19.52 ± 0.16 a	9.19 ± 0.25 a	2.06 ± 0.04 ab	548.19 ± 28.95 ab	20.48 ± 0.11 ab
	BGN	16.02 ± 0.54 c	8.53 ± 0.10 bc	1.98 ± 0.04 bc	405.07 ± 16.97 de	17.39 ± 0.47 d
	D	21.42 ± 0.36 b	10.54 ± 0.28 a	2.23 ± 0.02 b	615.46 ± 9.44 b	24.76 ± 0.37 b
	DN	16.63 ± 0.46 d	8.71 ± 0.17 e	1.94 ± 0.03 d	512.58 ± 8.37 c	21.82 ± 0.12 d
	DG	23.80 ± 0.50 a	10.68 ± 0.22 a	2.39 ± 0.02 a	740.83 ± 31.61 a	27.32 ± 0.32 a
6	DGN	19.34 ± 0.51 c	9.77 ± 0.01 bc	2.14 ± 0.04 bc	595.52 ± 18.58 b	23.12 ± 0.29 c
	B	21.07 ± 0.39 b	10.18 ± 0.06 ab	2.16 ± 0.04 bc	595.24 ± 7.41 b	24.72 ± 0.10 b
	BN	16.28 ± 0.38 d	8.98 ± 0.14 de	1.91 ± 0.02 d	497.32 ± 11.87 c	20.42 ± 0.35 e
	BG	22.41 ± 0.21 b	10.71 ± 0.24 a	2.35 ± 0.05 a	705.73 ± 20.95 a	26.70 ± 0.09 a
	BGN	18.09 ± 0.59 c	9.42 ± 0.09 cd	2.10 ± 0.04 c	519.36 ± 15.72 c	21.59 ± 0.46 d
	D	24.42 ± 0.36 bc	12.00 ± 0.28 a	2.58 ± 0.02 b	880.77 ± 9.44 c	31.44 ± 0.28 b
	DN	18.05 ± 0.46 f	9.32 ± 0.17 e	2.15 ± 0.03 e	662.63 ± 11.87 e	26.95 ± 0.12 d
	DG	26.80 ± 0.70 a	12.48 ± 0.22 a	2.83 ± 0.03 a	1051.12 ± 37.27 a	34.10 ± 0.31 a
9	DGN	22.92 ± 0.26 d	11.02 ± 0.02 bc	2.33 ± 0.02 d	777.49 ± 18.81 d	29.21 ± 0.29 c
	B	24.02 ± 0.39 cd	11.43 ± 0.06 b	2.47 ± 0.04 c	860.55 ± 7.41 c	31.39 ± 0.10 b
	BN	17.50 ± 0.38 f	10.25 ± 0.14 d	2.12 ± 0.02 e	648.89 ± 20.83 e	25.55 ± 0.35 e
	BG	25.65 ± 0.17 ab	12.36 ± 0.24 a	2.75 ± 0.04 a	971.37 ± 28.22 b	33.48 ± 0.11 a
	BGN	20.23 ± 0.56 e	10.58 ± 0.09 cd	2.32 ± 0.06 d	755.67 ± 16.28 d	27.68 ± 0.47 d

Note: D (drill sowing); DN (drill sowing + NaCl); B (broadcast sowing); BN (broadcast sowing + NaCl); DG (drill sowing + GR24); BG (broadcast sowing + GR24); DGN (drill sowing +GR24 + NaCl); BGN (broadcast sowing + GR24 + NaCl). Data are presented as mean ± standard error of three replicates. Different lowercase letters within a column indicate significant differences among treatments at *p* < 0.05.

## Data Availability

Data may be obtained through the corresponding author for reasonable reasons.

## Abbreviations

The following abbreviations are used in this manuscript:

ROS	reactive oxygen species
MDA	malondialdehyde
SLs	strigolactones
POD	peroxidase
SOD	superoxide dismutase
GSH	glutathione
AsA	ascorbic acid
ETR	electron transport rate
NPQ	non-photochemical quenching coefficient
PPO	polyphenol oxidase
PAL	phenylalanine ammonia-lyase
ELISA	enzyme-linked immunosorbent assay
ANOVA	analysis of variance
Sm	sowing method
Rf	regulatory factor
Pn	net photosynthetic rate
Gs	stomatal conductance
Ci	intercellular CO_2_ concentration
Tr	transpiration rate
Fo	initial fluorescence
Fv/Fm	maximum photochemical efficiency of PSII
ΦPSII	actual quantum yield of PSII
qP	photochemical quenching coefficient
H_2_O_2_	hydrogen peroxide
O·^−^	superoxide anion generation rate
APX	ascorbate peroxidase
CAT	catalase
GSSG	oxidized glutathione
GR	glutathione reductase
MDHAR	monodehydroascorbate reductase
DHAR	dehydroascorbate reductase
